# An alternative method of indexation in aortic stenosis: height-adjusted effective orifice area: An observational prospective study

**DOI:** 10.1186/s12947-023-00314-8

**Published:** 2023-08-22

**Authors:** Sergio Gamaza-Chulián, Fátima González-Testón, Enrique Díaz-Retamino, Francisco M. Zafra-Cobo, Eva González-Caballero

**Affiliations:** Cardiology Department, Jerez Hospital, Carretera Circunvalación s/n 11407, Jerez de la Frontera, Cádiz, Spain

**Keywords:** Aortic stenosis, Aortic valve area, Echocardiography, Prognosis

## Abstract

**Background:**

Although indexing effective orifice area (EOA) by body surface area (BSA) is recommended, this method has several disadvantages, since it corrects by acquired fatty tissue. Our aim was to analyze the value of EOA normalized by height for predicting cardiovascular outcome in patients with aortic stenosis (AS).

**Methods:**

Patients with AS (peak velocity > 2 m/s) evaluated in our echocardiography laboratory between January 2015 and June 2018 were prospectively enrolled. EOA was indexed by BSA and height. A composite primary endpoint was defined as cardiac death or aortic valve replacement. A receiver operating characteristic curve was plotted to determine the best cutoff value of EOA/height for predicting cardiovascular events.

**Results:**

Four-hundred and fifteen patients were included (52% women, mean age 74.8 ± 11.6 years). Area under the curve was similar for EOA/BSA (AUC 0.75, *p* < 0.001) and EOA/height (AUC 0.75, *p* < 0.001). A cutoff value of 0.60 cm^2^/m for EOA/height had a sensitivity of 84%, specificity of 61%, positive predictive value of 60% and negative predictive value of 84%. One-year survival from primary endpoint was significantly lower in patients with EOA/height ≤ 0.60 cm^2^/m (48 ± 5% vs 91 ± 4%, log-rank *p* < 0.001) than EOA/height > 0.60 cm^2^/m. The excess of risk of cardiovascular events seen in univariate analysis persists even after adjustment for other demonstrated adverse prognostic variables (HR 5.91, 95% CI 3.21–10.88, *p* < 0.001). In obese patients, there was an excess of risk in patients with EOA/height < 0.60 cm2/m (HR 10.2, 95% CI 3.5–29.5, *p* < 0.001), but not in EOA/BSA < 0.60 cm^2^/m^2^ (HR 0.14, 95% CI 0.14–1.4, *p* = 0.23).

**Conclusions:**

We could identify a subgroup of patients with AS at high risk of cardiovascular events. Consequently, we recommend using EOA/height as a method of indexation in AS, especially in obese patients, with a cutoff of 0.60 cm2/m for identifying patients with higher cardiovascular risk.

**Graphical Abstract:**

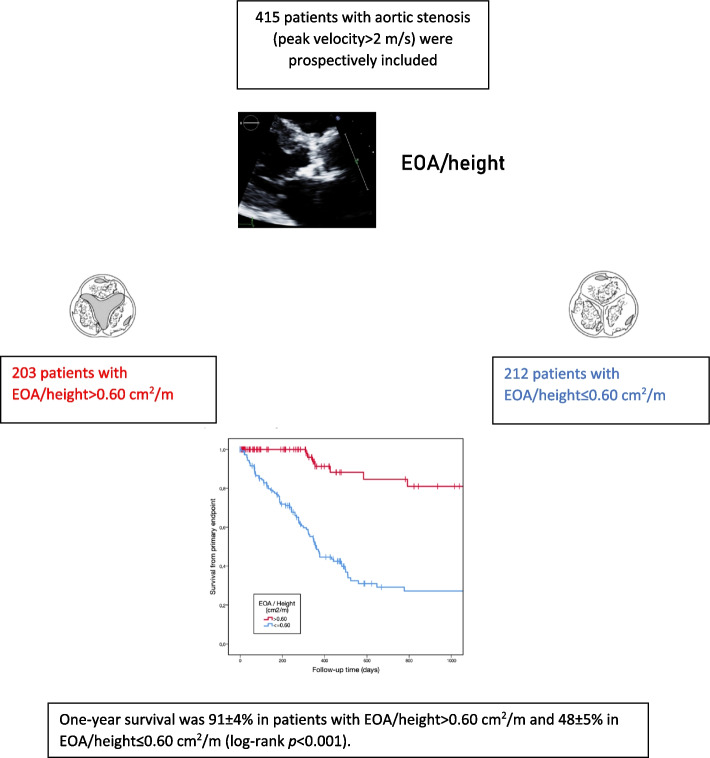

## Introduction

Aortic stenosis (AS) is the most common primary valve lesion in Europe and North America, and its prevalence continues rising as a consequence of the ageing population [1]. Echocardiography is an essential tool to confirm the diagnosis and severity of AS [2, 3], and the main parameters that current guidelines of the European Society of Cardiology recommends to evaluate are mean pressure gradient, peak transvalvular velocity and effective orifice area (EOA) [4].

Indexing EOA by body surface area (BSA) represents an intuitively convincing and widely adopted method to adjust for differences in body size. However, normalization for BSA has been criticized in patients with obesity where it corrects not only by body size but also for the acquired fatty tissue [5, 6]. In addition to this, scientific data to support this approach are scarce [7] and existing studies do not provide information about predictive accuracy for the occurrence of AS-related events.

Our aim was to analyze the value of EOA normalized by height for predicting cardiovascular outcome in patients with AS, and to identify the best EOA/height cutoff for prediction of high risk of cardiovascular events during follow-up.

## Material and methods

### Patient population

We prospectively included 415 patients between January 2015 and June 2018 with valvular native AS (peak velocity > 2 m/s). The exclusion criteria were: age under 18 years old, suboptimal acoustic window, concomitant moderate or severe aortic regurgitation, moderate or severe mitral or tricuspid valvular disease, subvalvular o supravalvular aortic stenosis (defined as velocity higher than 1.5 m/s), ascending thoracic aorta diameter less than 25 mm, congenital heart disease (except bicuspid aortic valve) and previous aortic valve surgery. We also enrolled prospectively 66 patients between January 2019 and September 2019 to create a validation group.

The study protocol was approved by the Ethics Committee of our center. All the participants gave their consent to participate in the study.

### Clinical data

Clinical data included age, sex, hypertension, diabetes, history of smoking, hypercholesterolemia, height, weight, chronic renal failure and coronary heart disease. Patients were carefully screened for the presence of symptoms attributable to AS: angina, syncope or dyspnoea using the New York Heart Association functional classification. Body surface area (BSA, m^2^) was calculated using the Dubois formula [8].

Clinical decisions on medical management were made by the referring physician based on AS severity, left ventricular function, and symptomatic status, according to guidelines [4].

### Echocardiographic examination

Two-dimensional transthoracic echocardiographic and Doppler studies were obtained with clinical ultrasound machines equipped with 2.5 to 3.5 MHz transducers (iE33 Phillips Medical Systems, The Best, The Netherlands). All tests were conducted by two experienced sonographers. Blood pressure was measured at the time of the echocardiographic evaluation.

Parasternal long axis view with zoom was used for measuring the aortic annulus diameter in early systole. Using the pulsed Doppler in the left ventricular outflow tract, placing the sample volume 1 cm below the aortic valve, the time-velocity integral (TVI) was obtained. Stroke volume was then calculated assuming a circular shape of the left ventricular outflow tract. Continuous wave Doppler recording of flow through the valve was performed from the 5-chambers and right parasternal windows to record maximal instantaneous and mean pressure gradients across the aortic valve.

EOA was calculated using the continuity equation. An indexed EOA was estimated as EOA/body surface area (EOA/BSA, cm^2^/m^2^). EOA was also normalized by height (EOA/height, cm^2^/m).

Mean transvalvular pressure gradient was obtained with the use of the modified Bernoulli equation. A Doppler velocity index (DVI), a simplification of the continuity equation, was calculated as TVI of left ventricular outflow tract/TVI of aortic jet.

All measurements represent an average of 3 cardiac cycles for patients in sinus rhythm and at least 6 cycles if the patient was in a different rhythm than sinus one. In any case, the estimation of extrasystolic beat was always avoided. Doppler recordings were performed at a sweep speed of 150 mm/s.

Dobutamine stress echocardiogram was performed when EOA calculated by continuity equation was less than 1.0 cm^2^, aortic transvalvular mean gradient inferior than 40 mmHg and left ventricular ejection fraction less than 40% [9]. A low-dose dobutamine infusion was begun after the baseline study at 5 µg/kg body weight/minute up to 20 µg/kg/min, titrated upwards at steps of 5 µg/kg/min every 5 min [10]. Doppler spectrograms of left ventricular outflow tract and AS jet velocity were obtained within the last 2 min of each dose. Blood pressure was monitored using automatic sphygmomanometer. Beta-blocker therapy was suspended 24 h before the index examination.

The systolic time intervals of flow through the aortic valve were measured using the velocity curve from the continuous wave Doppler recording in apical view: ejection time (ET), acceleration time (AT) and AT/ET ratio, as we described previously [11].

For classification purpose, in patients with left ventricular ejection fraction above 50%, mild AS was defined as a peak velocity below 3.0 m/s; moderate AS whether peak velocity was between 3.0 and 4.0 m/s; whilst severe AS was defined when peak velocity was greater than 4.0 m/s. In patients with left ventricular dysfunction, “classical” low-flow low gradient severe AS was defined by a peak velocity > 4 m/s or a mean gradient > 40 mmHg and an aortic valve area that not exceed 1.0 cm^2^ after stress echocardiography. A “paradoxical” low flow-low gradient severe AS was defined as an EOA smaller than 1.0 cm^2^, mean gradient less than 40 mmHg, left ventricular ejection fraction above 50% and stroke volume index ≤ 35 ml/m^2^.

### Outcomes

The primary endpoint was a combined endpoint of cardiovascular death and aortic valve replacement. In patients who underwent aortic valve implantation, we used the implantation date to compute the length of follow-up. We also recorded cardiac death, percutaneous and surgical aortic valve replacement. Cardiac death includes death resulting from sudden cardiac death, death due to heart failure, stroke or cardiovascular procedures. Outcome data were retrospectively obtained from patient visits or records, telephone interview, or death certificates when applicable.

### Statistical analysis

Continuous variables were tested for normality using the Shapiro–Wilk test. Data were expressed as mean ± standard deviation for continuous variables, and were compared using the unpaired *t* test. Categorical variables were expressed as percentages and were compared using chi-square analysis or the Fisher exact test. A receiver-operating characteristic curve was plotted to determine the optimal cutoff value of EOA/height for predicting one-year primary endpoint in patients with AS. The best cutoff value was determined as the value providing a balance between sensitivity and specificity. A receiver-operating characteristic curve of EOA/BSA was also plotted. The area under the curve (AUC) was calculated. The calculated cut-off was then tested in the validation cohort.

Kaplan–Meier analysis were performed by using the log-rank test to compare survival rates between the groups. Univariate and multivariable analyses of time to events were performed using Cox proportional hazard models. The variables entered into the model were sex, age, left ventricular ejection fraction, peak aortic velocity, indexed left ventricular mass, symptomatic status and EOA/height, based on their demonstrated prognostic value in AS.

To study the predictive value of EOA/height AND EOA/BSA in obese patients, univariable Cox model testing the impact of EOA/height ≤ 0.6 cm^2^/m and EOA/BSA ≤ 0.60 cm^2^/m^2^ on primary endpoint in patients with obesity (BMI ≥ 30 kg/m^2^).

We aimed also at identifying if there was a difference in the prognostic value of EOA/height ≤ 0.6 cm^2^/m in subgroups of patients. Hence, a first-order interaction term (the product of EOA/height ≤ or > 0.6 cm^2^/m and different categories of subgroups) was systematically included in a Cox multivariable model including EOA/height ≤ or > 0.6 cm^2^/m and the categories of each subgroup of patients in the whole cohort of patients. A significant interaction was considered in case of a *p* value for the interaction variable < 0.05. Univariable Cox model testing the impact of EOA/height ≤ or > 0.6 cm^2^/m on primary endpoint were obtained thereafter in each category of the subgroup of patients.

Differences were considered significant at *p* values < 0.05. For data analysis, the statistical program SPSS version 19.0 (SPSS Inc., Chicago, Illinois) was used.

## Results

A final sample of 415 patients were enrolled, of which 45 (11%) had mild AS, 181 (44%) moderate AS, 162 (39%) severe AS, 20 (5%) classical low flow-low gradient severe AS and 7 (2%) “paradoxical” low-flow low gradient severe AS. Mean age was 74.8 ± 11.6 years, with 52% women and a body mass index of 29.4 ± 5.0 kg/m^2^. Diabetes prevalence was 44%, arterial hypertension 80%, obesity (BMI ≥ 30 kg/m^2^) 42% and coronary artery disease 42%. Degenerative calcification was the most common cause of AS (92%), followed by bicuspid aortic valve (6%) and rheumatic disease (2%). Overall, aortic peak velocity was 3.77 ± 0.77 m/s, mean gradient 36.3 ± 15.5 mmHg, EOA 1.01 ± 0.35 cm^2^ and left ventricular ejection fraction 61.1 ± 10.7%.

Validation group was composed of 4 patients (6%) with mild AS, 25 (53%) moderate AS, 36 (29%) severe AS and 1 (1%) patient with “classical” low flow-low gradient severe AS.

### Receiver operating characteristics analysis

**Fig. 1:**
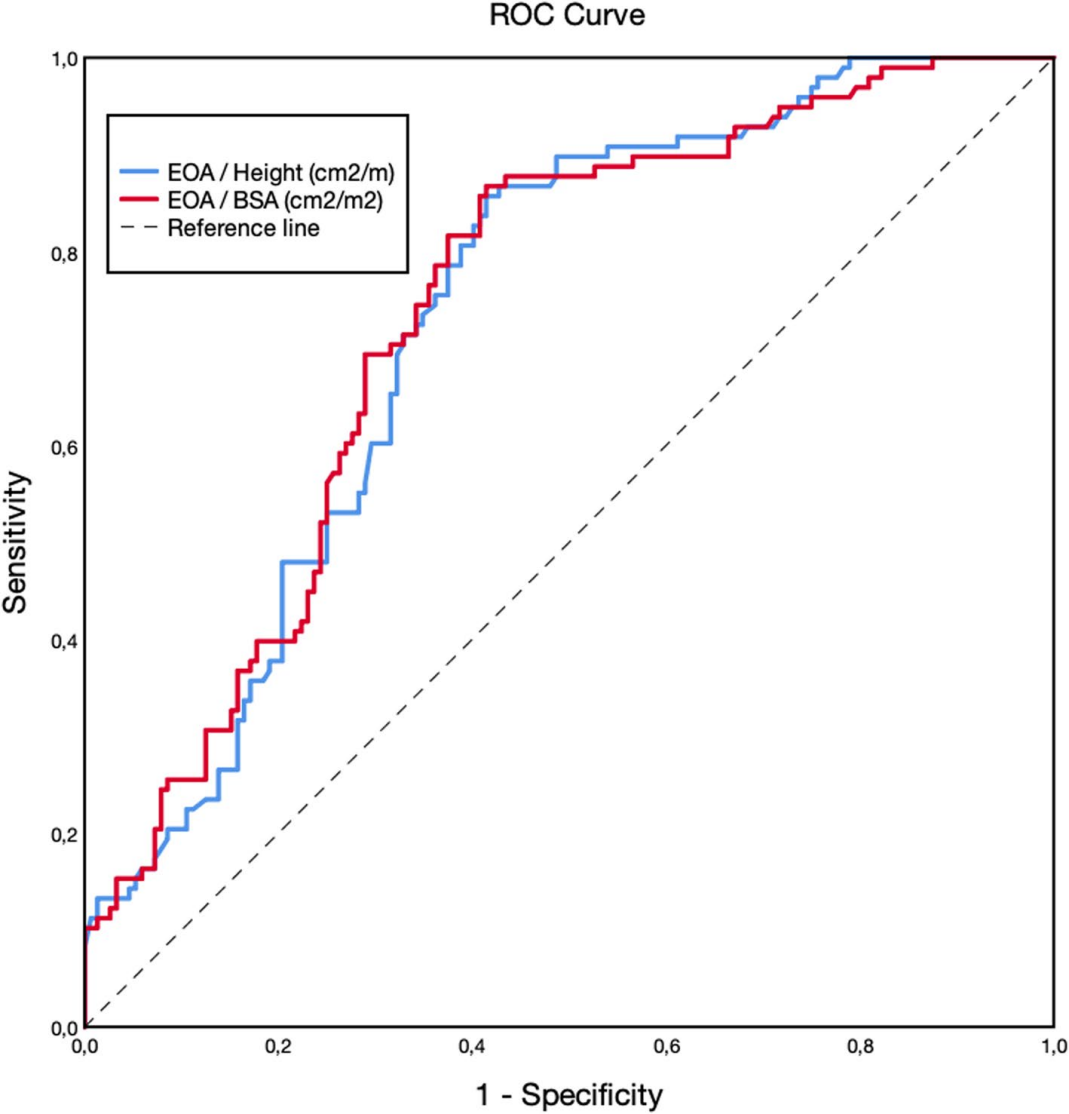
Receiver operating characteristics curves for predicting cardiovascular events

Receiver operating characteristics curves (Fig. 1) showed that both EOA/height and EOA/BSA could significantly predict one-year cardiovascular outcomes. Both variables had similar AUC: EOA/height (AUC 0.75, *p* < 0.001) and EOA/BSA (AUC 0.75, *p* < 0.001). AUC was also similar for predicting 6-month cardiovascular events in EOA/height (AUC 0.74, *p* < 0.001) and EOA/BSA (AUC 0.74, *p* < 0.001), and two-year outcomes (AUC 0.90, *p* < 0.001 and AUC 0.91, *p* < 0.001, respectively).

Table 1 summarizes the best cut-off values for EOA/height, balancing sensitivity and specificity for severe AS as well as those cut-offs values with 100% sensitivity and specificity for predicting the primary endpoint in the derivation group. Using a cutoff value of 0.60 cm^2^/m, EOA/height had a sensitivity of 84% (CI 95%, from 77 to 91%), specificity of 61% (CI 95%, from 53 to 69%), positive predictive value of 60% (CI 95%, from 52 to 68%) and negative of 84% (CI 95% from 78 to 92%), whilst using the guideline-recommended cutoff value of 0.60 cm^2^/m^2^, EOA/BSA had a sensitivity of 92% (CI 95% from 84 to 98%), specificity of 38% (CI 95% from 30 to 46%), positive predictive value of 51% (CI 95% from 43 to 51%) and negative predictive value of 87% (CI 95% from 80 to 94%).

**Table 1:** ROC analysis: different cutoff values of EOA/height (cm^2^/m) for predicting cardiovascular outcomes

Cutoff	Sens. (%)	Spec.(%)	Accuracy(%)	PPV(%)	NPV(%)
0.80	100	23	69	48	100
0.60	84	61	71	60	84
0.31	11	100	63	100	61

*AUC* Area under the curve, *Sens* Sensitivity, *Spec* Specificity, *PPV* Positive Predictive Value, *NPV* Negative Predictive Value

In the validation group, using a cut-off of 0.60 cm^2^/m, EOA/height had a sensitivity of 77%, a specificity of 70%, a positive predictive value of 62% and negative predictive value of 82% for severe AS. However, the cutoff of 0.60 cm2/m2 for EOA/BSA had a sensitivity of 88%, specificity of 50%, positive predictive value of 53% and negative predictive value of 87%.

### Outcomes

Derivation group patients were divided into two groups according to EOA/height value: 212 patients with EOA/height equal to or lower than 0.60 cm^2^/m and 203 patients with EOA/height higher than 0.60 cm^2^/m. Baseline characteristics of patients are compared in Table 2. Patients with EOA/height ≤ 0.60 cm^2^/m showed more severe echocardiographic parameters (Table 3).

**Table 2:** Baseline characteristics according to EOA/height value

Variable	EOA/height>0.60 cm^2^/m (*n*=203)	EOA/height≤0.60 cm^2^/m (*n*=212)	*p*
Women	103 (51%)	114 (54%)	0.54
Hypertension	165 (82%)	165 (78%)	0.42
Diabetes	89 (44%)	93 (44%)	0.90
Age (years)	73.5±13.2	76.1±9.7	0.02
BMI (kg/m^2^)	30.3±4.7	28.5±5.1	<0.001
Coronary disease	73 (36%)	84 (40%)	0.75
Degenerative	185 (91%)	197 (93%)	0.32
CrCl<30 ml/min/m^2^	26 (13%)	27 (13%)	0.92
Symptomatic	107 (53%)	172 (81%)	<0.001
Heart rate (bpm)	75±14	73±15	0.23

*EOA* Effective Orifice Area, *BMI* Body Mass Index, *CrCl* Creatinine Clearance, *bpm* beats per minute

**Table 3:** Echocardiographic characteristics according to EOA/height value

Variable	EOA/height>0.60 cm^2^/m (*n*=203)	EOA/height≤0.60 cm^2^/m (*n*=212)	*p*
Peak velocity (m/s)	3.20±0.54	4.31±0.52	<0.001
Mean gradient (mmHg)	24.9±8.9	47.2±12.3	<0.001
EOA (cm^2^)	1.30±0.26	0.73±0.15	<0.001
EOA/BSA (cm^2^/m^2^)	0.69±0.15	0.41±0.08	<0.001
EOA/height (cm^2^/m)	0.80±0.16	0.46±0.09	<0.001
LVEF (%)	63.2±8.3	59.1±12.2	<0.001
Indexed LVM (g/m^2^)	114.5±31.5	141.5±36.9	<0.001
AT/ET	0.26±0.06	0.36±0.06	<0.001
DVI	0.37±0.09	0.22±0.05	<0.001

*EOA* Effective Orifice Area, *BSA* Body Surface Area, *LVEF* Left Ventricular Ejection Fraction, *LVM* Left Ventricular Mass, *AT* Acceleration Time, *ET* Ejection Time, *DVI* Doppler Velocity Index

Complete follow-up was achieved in 100% of the sample. Median follow-up was 322 days (from 1 to 1538 days), without significant differences according to EOA/height group (385 ± 448 vs 352 ± 358, *p* = 0.52).

**Fig. 2:**
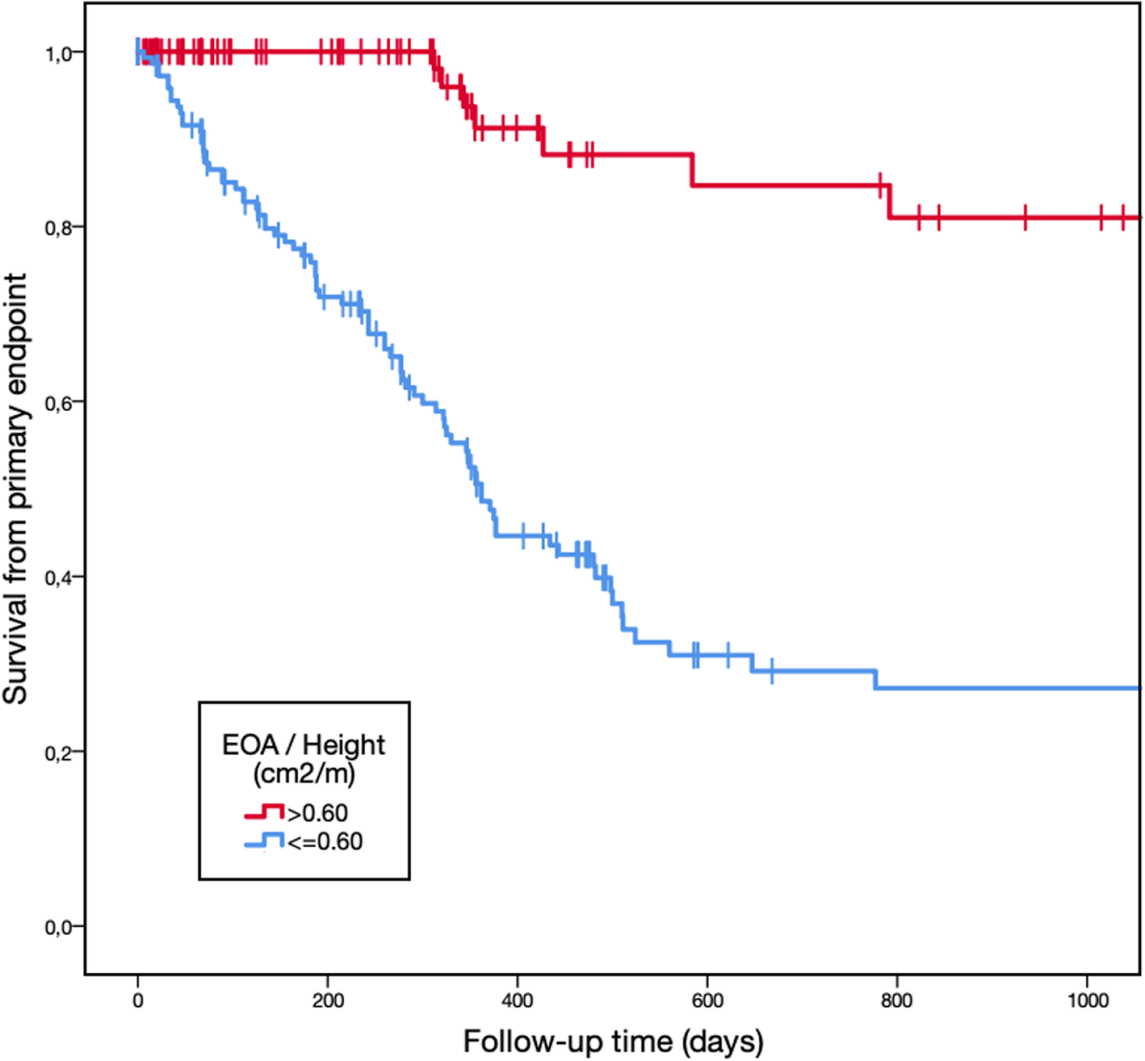
Kaplan-Meier plots showing risk for cardiovascular combined endpoint according to EOA/height

Primary endpoint was reached in 116 patients (28%) during follow-up. There were 57 deaths, of which 33 were from cardiovascular causes. Aortic valve replacement was performed in 86 patients during follow-up. Survival from primary endpoint is presented in Fig. 2. There were more combined cardiovascular events in patients with EOA/height ≤ 0.60 cm^2^/m (60% vs 16%, *p* < 0.001), more cardiovascular deaths (18% vs 3%, *p* < 0.001), although global mortality did not reach statistical differences (23% vs 17%, *p* = 0.27).

Similarly, there were more combined cardiovascular events in patients with EOA/BSA ≤ 0.60 cm2/m2 (47% vs 16%, *p* < 0.001), although cardiovascular death did not reach statistical difference (13% vs 5%, *p* = 0.05). Global mortality did not reach significant differences either (25% vs 15%, *p* = 0.12).

One-year survival was 91 ± 4% in patients with EOA/height > 0.60 cm^2^/m and 48 ± 5% in EOA/height ≤ 0.60 cm^2^/m (log-rank *p* < 0.001). Moreover, two-year survival was 85 ± 6% in EOA/height > 0.60 cm^2^/m and 27 ± 5% in EOA/height ≤ 0.60 cm^2^/m (log-rank *p* < 0.001).

### Obese patients

One-hundred and seventy-five patients had obesity (BMI ≥ 30 kg/m^2^) in our sample (42% of the patients), of which 54% were women, 21% had mild, 45% moderate and 34% severe AS. Mean age was 75.1 ± 8.3 years and BMI was 34.1 ± 3.4 kg/m^2^. There was an excess of risk in patients with EOA/height < 0.60 cm2/m (HR 10.2, 95% CI 3.5–29.5, *p* < 0.001), but not in EOA/BSA < 0.60 cm^2^/m^2^ (HR 0.14, 95% CI 0.14–1.4, *p* = 0.23).

Sensitivity of EOA/height < 0.60 cm^2^/m for predicting cardiovascular events in obesity was 83%, specificity 70%, negative predictive value 88% and positive predictive value of 62%. Youden Index was 53.3%. AUC of EOA/height in obesity was 0.71 (CI 0.63–0.79, *p *< 0.001).

In obese patients with EOA/BSA < 0.60 cm2/m2, sensitivity was 95%, specificity 32%, negative predictive value 92% and positive predictive value of 45%. Youden Index was 27%. AUC of EOA/BSA in obese patients was 0.69 (0.61–0.77, *p* < 0.001).

### Multivariate analysis

The excess of risk of cardiovascular events in univariate analysis in patients with EOA/height ≤ 0.60 cm2/m (HR 4.61, 95% confidence interval (95% CI) 2.66–7.98, *p* < 0.001) was also observed after adjustment by age, sex, left ventricular ejection fraction, symptomatic status, indexed left ventricular mass and peak aortic velocity (HR 5.91, 95% CI 3.21–10.88, *p* < 0.001) (Table 4). However, EOA/BSA was not significant after adjustment by those variables (HR 1.41, 95% CI 0.41–3.10, *p* = 0.24).

**Table 4:** Relative risk of cardiovascular events during follow-up associated with EOA/height

	HR (95% CI)	*P*
EOA/height≤0.6 cm^2^/m		
Unadjusted	4.61 (2.66-7.98)	<0.001
Model 1	4.91 (2.79-8.64)	<0.001
Model 2	4.64 (2.62-8.19)	<0.001
Model 3	5.91 (3.21-10.88)	<0.001

95% CI: 95% Confidence Interval; EOA: Effective Orifice Area

Model 1 is adjusted for sex, age, aortic peak velocity and symptomatic status

Model 2 is adjusted for sex, age, aortic peak velocity, symptomatic status and left ventricular ejection fraction

Model 3 is adjusted for sex, age, aortic peak velocity, symptomatic status, left ventricular ejection fraction and indexed left ventricular mass

**Fig. 3:**
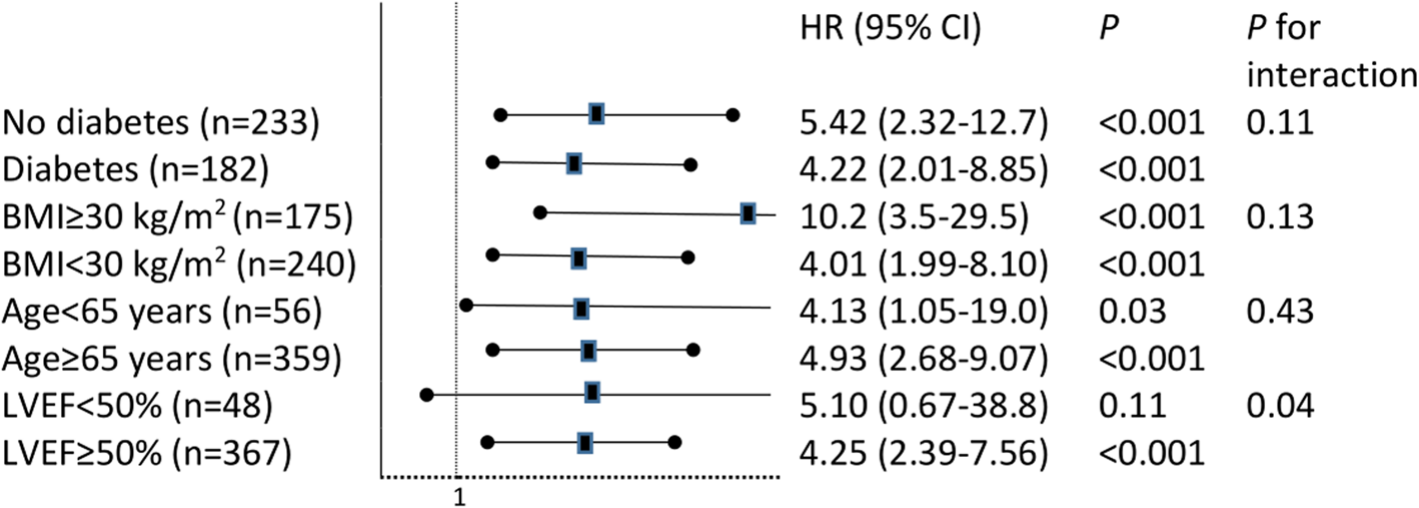
Risk of cardiovascular events associated with EOA/height=0.60 cm2/m in subgroups of patients with AS. HR: Hazard Ratio; CI: Confidence Interval; BMI: Body Mass Index; LVEF: Left Ventricular Ejection Fraction.

The impact of EOA/height ≤ 0.60 cm2/m was consistent in different subgroups, except for left ventricular ejection fraction where a significant interaction was found (p for interaction = 0.04, Fig. 3).

## Discussion

To the best of our knowledge, this is one of the few studies to specifically assess the outcome implications of EOA indexed to height. According to our results, EOA/height predicts the occurrence of cardiovascular events after AS diagnosis. Furthermore, the impact of EOA/height on the risk of cardiovascular events persists even after adjustment for other demonstrated adverse prognostic variables.

The definition of severe AS by current guidelines of the European Society of Cardiology (ESC) incorporates an EOA < 1 cm^2^, based on several studies that show that prognosis is impaired below this value [12–16]. It could be reasonable to adjust EOA to body size in order to remove morphometric differences. Although indexing EOA by BSA is recommended in current guidelines [17], outcome data to support this approach are scarce [18, 19], and the current EOA/BSA cutoff of 0.6 cm^2^/m^2^ increases the discrepancies between aortic valve area and gradients [20–23]. This misdiagnosis based on EOA/BSA is especially true in obese patients [5], where excess weight accounts for an increase in BSA and a decrease in indexed EOA [6].

In contrast, height may be considered a better parameter for adjusting EOA as it is not altered by excessive adipose tissue, remains practically unchanged during adulthood, and allows adjustment for body size differences between different nationalities [24].

Indeed, our data showed that outcome in patients with EOA was not influenced by BMI, that is, in our opinion, one of the strengths of the study.

*Vulesevic *et al. [24] showed that EOA/height better correlates with severe AS than EOA/BSA and established a cutoff value of 0.6 cm2/m for EOA/height, defining severe AS as an aortic valve area < 1 cm^2^. However, we preferred to use clinical outcome because it is recognized as the only endpoint available for defining severity [25]. 

The good predictive capacity for cardiovascular events of EOA/height was also shown by *Tribouilloy *et al. [21], with excess event rate below 0.45 cm2/m. However, we found a higher cutoff value, probably because we included both patients with symptoms and with impaired systolic function.

Although it is not the objective of the study, we failed to demonstrate a higher predictive accuracy of EOA/height compared to EOA/BSA, with similar area under the curve for predicting cardiovascular events. Nevertheless, we believe that EOA/height has several advantages over EOA/BSA: 1) EOA/BSA may overestimate the severity of AS in obese patients [26], whilst our data showed that EOA/height was not influenced by body mass index; 2) Indeed, we demonstrated that EOA/height has higher predictive value in obese patients, who account for an important percentage of the general population; 3) BSA can change throughout life whilst height remains practically unchanged during adulthood.

The main limitation of our study is, in our opinion, the small sample size, which could make it difficult to obtain statistically significant differences in some results. Secondly, sample data were obtained from a single hospital. Although it may be argued that valve replacement is an end point that is arbitrarily determined during the natural history of the disease, it is noteworthy that referring physicians based their decisions on EOA, peak velocity, mean gradients and symptomatic status, being unaware of EOA/height values. Another probable limitation is the high prevalence of obese patients in our sample, that could influence the results, especially in EOA/BSA. Besides, validation group sample was small. Finally, it would have been interesting to analyze cardiovascular events in asymptomatic patients, but we did not have an adequate sample size to obtain significant results.

In conclusion, the present study shows that normalization of EOA for height is useful for risk stratification, since we could identify a subgroup of patients at high risk of cardiovascular events. Therefore, EOA/height represents a variable that provides additional information to EOA/BSA in AS, and the cut-off of 0.6 cm^2^/m provides a predictive accuracy for the occurrence of AS-related events.

## Data Availability

The datasets used and/or analysed during the current study are available from the corresponding author on reasonable request.
